# Ecological correlates of cranial evolution in the megaradiation of dipsadine snakes

**DOI:** 10.1186/s12862-023-02157-3

**Published:** 2023-09-08

**Authors:** Gregory G. Pandelis, Michael C. Grundler, Daniel L. Rabosky

**Affiliations:** 1 Department of Ecology and Evolutionary Biology, University of Michigan, Ann Arbor, Michigan 48109 USA; 2 Museum of Zoology, University of Michigan, Ann Arbor, Michigan 48109 USA; 3 Amphibian and Reptile Diversity Research Center, Department of Biology, University of Texas at Arlington, Arlington, Texas 76019 USA

**Keywords:** Dipsadinae, Adaptive radiation, Ecomorphology, Geometric morphometrics, Skull morphology, Morphological diversification

## Abstract

**Background:**

Dipsadine snakes represent one of the most spectacular vertebrate radiations that have occurred in any continental setting, with over 800 species in South and Central America. Their species richness is paralleled by stunning ecological diversity, ranging from arboreal snail-eating and aquatic eel-eating specialists to terrestrial generalists. Despite the ecological importance of this clade, little is known about the extent to which ecological specialization shapes broader patterns of phenotypic diversity within the group. Here, we test how habitat use and diet have influenced morphological diversification in skull shape across 160 dipsadine species using micro-CT and 3-D geometric morphometrics, and we use a phylogenetic comparative approach to test the contributions of habitat use and diet composition to variation in skull shape among species.

**Results:**

We demonstrate that while both habitat use and diet are significant predictors of shape in many regions of the skull, habitat use significantly predicts shape in a greater number of skull regions when compared to diet. We also find that across ecological groupings, fossorial and aquatic behaviors result in the strongest deviations in morphospace for several skull regions. We use simulations to address the robustness of our results and describe statistical anomalies that can arise from the application of phylogenetic generalized least squares to complex shape data.

**Conclusions:**

Both habitat and dietary ecology are significantly correlated with skull shape in dipsadines; the strongest relationships involved skull shape in snakes with aquatic and fossorial lifestyles. This association between skull morphology and multiple ecological axes is consistent with a classic model of adaptive radiation and suggests that ecological factors were an important component in driving morphological diversification in the dipsadine megaradiation.

**Supplementary Information:**

The online version contains supplementary material available at 10.1186/s12862-023-02157-3.

## Background

Understanding the dynamics and causes of ecological and morphological diversification during major radiations is a key challenge in evolutionary biology. The relationship between ecology and morphology is central to our ability to test the role of ecological opportunity and other factors in mediating lineage and phenotype diversification during such radiations [1–3]. However, the ecological basis of phenotypic variation – while readily identifiable with relatively simple phenotypic traits [4] – presents an acute challenge when considering complex, highly-integrated and/or modular morphological structures [5, 6] with multiple functional modalities, such as the vertebrate skull. Previous work on vertebrate skulls has revealed an unexpected complexity to the form-function-ecology relationship [7–9]. Obtaining a clear picture of how this highly intricate structure diversifies in parallel with ecological factors has the potential to illuminate how and why some vertebrate clades have become so much more diverse than others, even when those other lineages appear to have had similar ecological opportunity and biogeographic context.

**Fig. 1:**
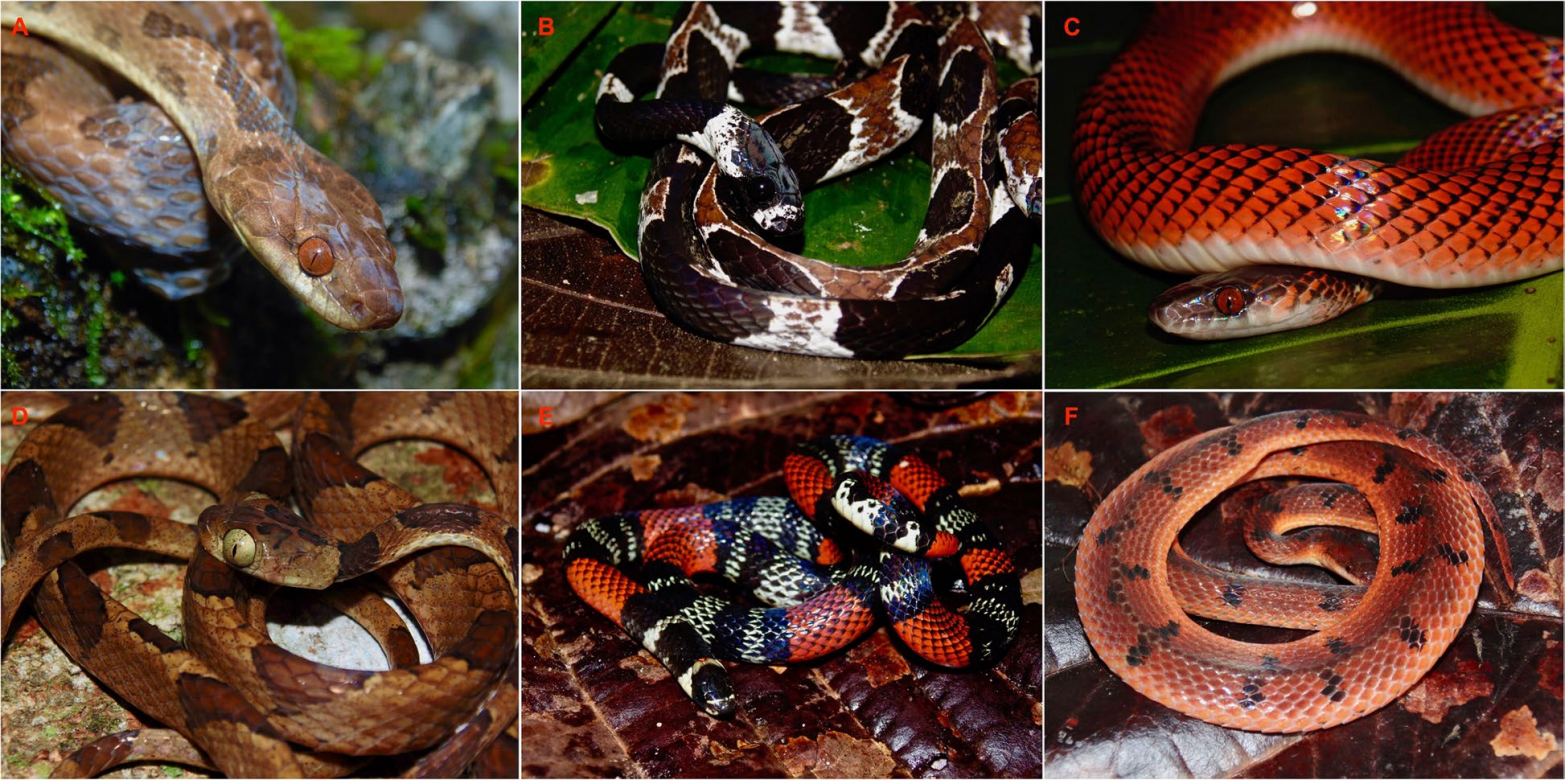
Diversity of neotropical dipsadine snakes. **A** – *Leptodeira septentrionalis*, a semi-arboreal frog specialist; **B** – *Dipsas catesbyi*, an arboreal snail-eating specialist; **C** – *Oxyrhopus melanogenys*, a terrestrial snake that typically feeds on reptiles; **D** – *Imantodes lentiferus*, an arboreal frog-eater; **E** – *Atractus elaps*, a small semi-fossorial snake that feeds on annelids; **F** – *Xenopholis scalaris*, a cryptic amphibian specialist, pictured here in a defensive flattening posture. See Fig. 2 for the skull morphology of these same six groups. (Photographs by G. Pandelis)

**Fig. 2:**
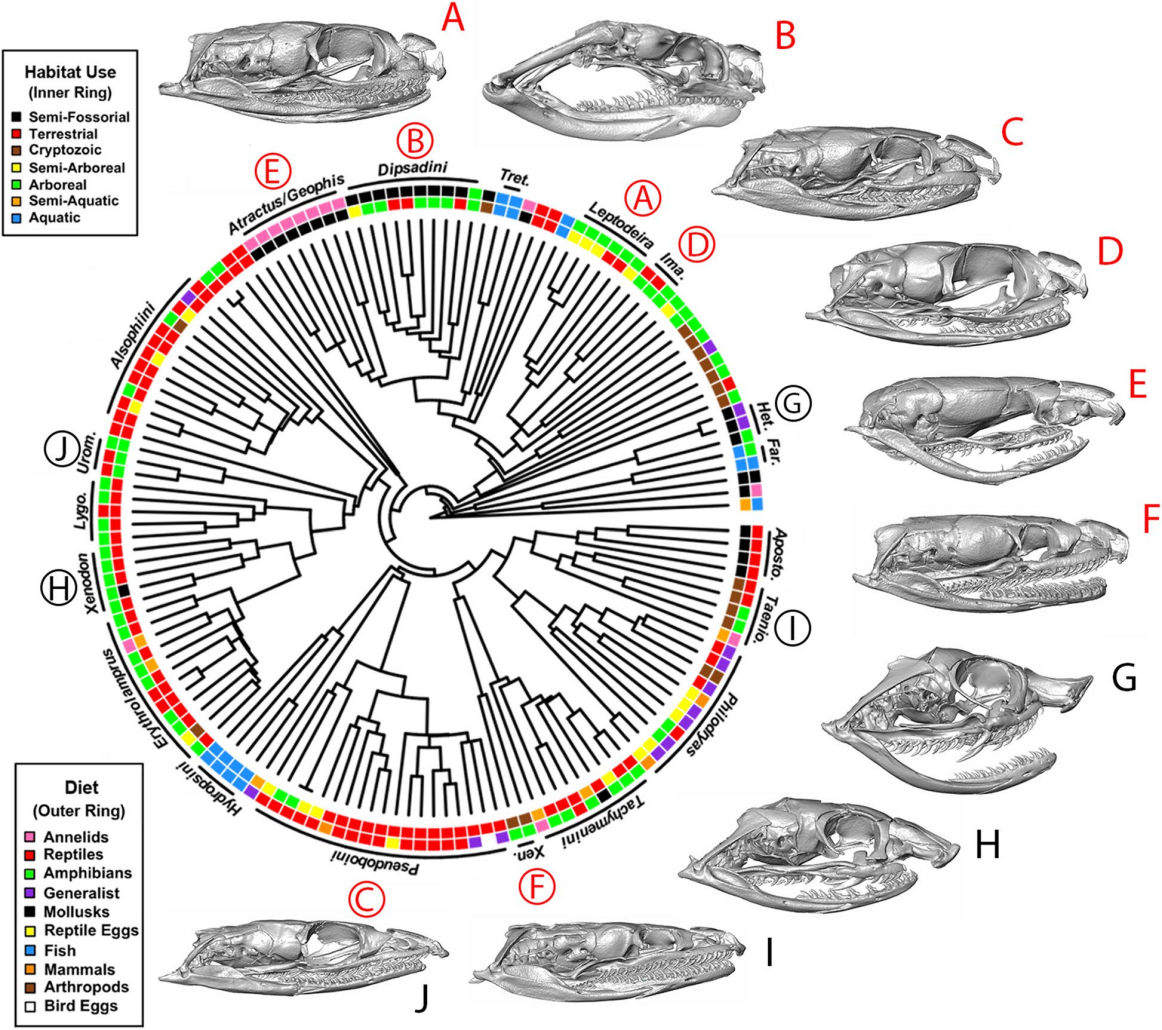
Ecological and morphological diversity across the dipsadine megaradiation. Habitat use and diet groups appear to have independent origins in multiple clades. Skull morphology is highly variable across the radiation, with conspicuous instances of probable convergence – for instance, note the remarkable morphological similarity between *Heterodon*, (G), an early-diverging North American dipsadine clade and *Xenodon*, (H), a deeply nested genus found in Central and South America. Position of skulls does not necessarily align with position in tree; circled letters correspond to the phylogenetic position of labeled skulls (uncircled). Skulls A-F correspond to the groups of the same letter pictured in Fig. 1. Abbreviations: Tret. – *Tretanorhinus*, Ima. – *Imantodes*, Het. – *Heterodon*, Far. – *Farancia*, Aposto. – *Apostolepis*, Taenio. – *Taeniophallus*, Xen. – *Xenopholis*, Lygo. – *Lygophis*, Urom. – *Uromacer*. A version of this figure with tip labels is available in the supporting material (Fig. S1)

In this article, we describe the evolutionary dynamics of the skull during one of the most diverse continental vertebrate radiations: the dipsadine snakes, which have undergone an extraordinary evolutionary explosion in the neotropics [10], diversifying into at least 806 species [11, 12] and a wide variety of ecological roles. In many cases, dipsadines account for over 50% of the species richness within neotropical snake communities [10, 13–15]. The (new world or western hemisphere) dipsadine megaradiation has received relatively little attention from a macroevolutionary perspective, despite its extreme ecological and morphological diversity (Figs. 1 and 2).

Dipsadines exhibit a vast range of dietary and habitat use profiles: diet can include snails [16], slugs [17], arthropods [18], annelids [19], bird eggs [20], mammals [21], birds [22], snakes and lizards [23], and fish [24] among other items, and individual species are characterized by varying degrees of specialization on those resources. Habitat use is also extremely variable in this clade, ranging from subterranean [25] to surface [26], arboreal [27], aquatic [28] and intermediate microhabitat use [29, 30]. Dipsadines encompass much of the ecological diversity seen across all snakes, and they have independently evolved many specialized ecologies that occur in distantly-related and biogeographically-separate snake lineages. Even within dipsadines, several specialized ecologies appear to have evolved multiple times (e.g. aquatic habitat use, molluscivory; Fig. 2). The recurrence of similar ecologies across the dipsadines suggests that they are a good system in which to test whether parallel phenotypic evolution has occurred in response to common selection pressures, as predicted by the ecological theory of adaptive radiation [1].

We focus on skull evolution because skull shape has important functional consequences for numerous ecological axes in vertebrates, including prey acquisition, handling, and ingestion [31, 32] habitat use [33], locomotion [34], sexual competition [35, 36], mate choice [37], and defense [38]. Numerous studies have investigated the dynamics of skull evolution with respect to ecology in various taxa, including mammals [7, 9, 35, 39, 40], birds [8, 41], ray-finned fishes [42, 43], and lizards [44, 45]. In general, these studies have shown a significant link between ecology and shape, although the importance of ecology in explaining morphological variation appears to vary across specific factors (e.g. diet, habitat, etc.) as well as across clades.

**Fig. 3:**
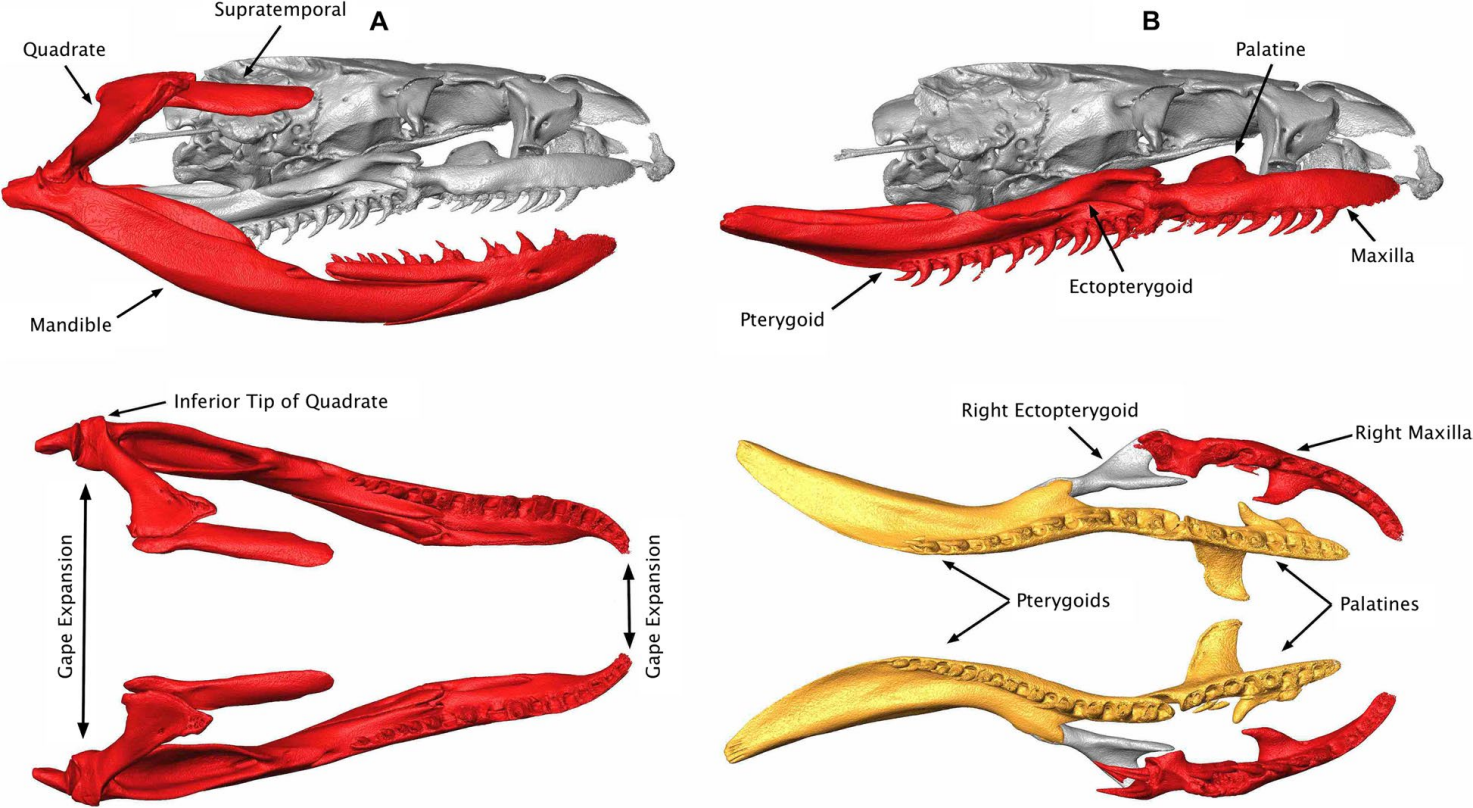
Trophic anatomy of the snake skull. **A** – A lateral view of the mandibular “chain” consisting of the supratemporal, quadrate, and mandible (top); a dorsal view of the mandibular chain (bottom). There are a total of 3 articulations here, all of which are kinetic and allow for expansion of the gape posteriorly (bottom). Gape expansion can also take place anteriorly due to a lack of mandibular connection or symphysis (bottom). **B** – The skull trophic “chain”, viewed laterally (top) and ventrally with other elements removed (bottom). The skull trophic chain consists of the lateral unit or maxilla (bottom – red) and the medial unit or palatal arch (bottom – gold). Note that the lateral and medial units are loosely bridged by the ectopterygoid; each side has the ability to move independently. Skull illustrated is a specimen of *Clelia clelia* (UMMZ 142639), a wide-ranging Central and South American terrestrial dipsadine that feeds primarily on other snakes

Numerous functional innovations distinguish the snake skull from that of their limbed (and limbless) “lizard” relatives, as well as from the skulls of most other vertebrates [46–50]. Perhaps most significantly, snake skulls are characterized by highly mobile elements (kinesis), partly accounting for their ability to consume large prey relative to their body size (Fig. 3) [38, 49, 51]. Snakes are thus able to exploit unique dietary niches that more gape-limited taxa are unable to capitalize on [38, 46]. These innovations may have provided snakes with a source of intrinsic ecological opportunity that allowed them to successfully radiate in novel environments [1, 3].

Many intriguing examples of convergent morphologies have been described within particular snake subclades [52], suggesting that skull morphology in snakes is, at least in some contexts, both evolutionarily labile and responsive to ecological pressures. One of the most iconic examples is found in snail eating specialists, namely, the new world dipsadine genera of *Dipsas* and *Sibon* and the old world genus *Pareas*. These geographically and taxonomically disparate groups are so similar in morphology that they were once thought to make up a single subfamily, although molecular and morphological data now show that they are separated by nearly 45 million years of independent evolution [12, 52]. Subsequent studies have corroborated the impact of diet on the evolution of snake head [53] and skull morphology [54–60] as well as the impact of habitat use [53, 59, 61–66], foraging mode and locomotion [61] and prey subjugation mode [60] in driving morphological diversification in the snake skull and head.

In our analyses of dipsadine skull evolution, we assess the breadth of morphological variation across the radiation in a phylogenetic comparative framework and we determine whether diet and habitat use are significant ecological predictors of this variation. A significant relationship between ecological traits and morphology would suggest that the extraordinary species richness of dipsadines has resulted in part from diversification along these ecological axes, thus suggesting a prominent role for adaptive radiation [1] in the assembly of neotropical snake faunas.

## Results

### PC axes

**Fig. 4:**
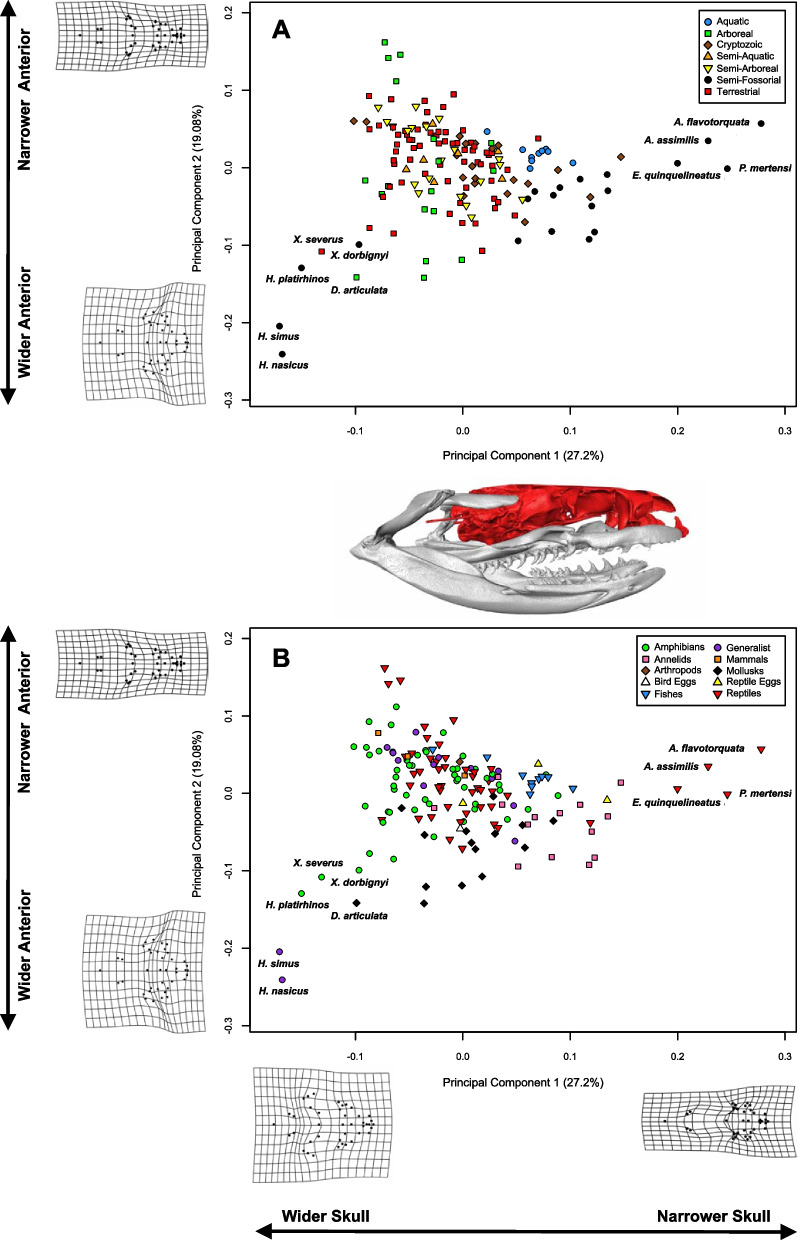
Principal components analysis of non-trophic module shape plotted with respect to habitat use (**A**) and diet (**B**). Shape deformations along PC1 and PC2 are illustrated with deformations of dorsal skull shape. With the exception of the outliers *Heterodon* and *Xenodon dorbignyi*, semi-fossorial snakes exhibit narrower skulls. Aquatic species tightly cluster in morphospace, to the right of most other groups, indicating highly similar morphology and relatively narrow skulls

In the non-trophic module analyzed for all 160 species, the first two PC axes accounted for 46.3% of shape variation, with PC1 accounting for 27.2% and PC2 accounting for 19.1% (Fig. 4, Fig. S2). Low PC1 values were associated with very broad, short skulls while high PC1 values were associated with very narrow, elongate skulls. PC2 tracked the extent to which the anterior portion of the skull was expanded and the extent to which the “snout” was elongated. Low PC2 values were associated with narrow anterior portions of the skull and elongate snouts while high PC2 values were associated with a severely expanded anterior region and short snout (Fig. 4, Fig. S2). Descriptions of the PC axes for the remaining skull modules can be found in Additional file 4: Appendix S4 of the supporting material.

### Description of skull morphospace

Within the non-trophic module, semi-fossorial snakes tend to cluster towards the right on PC1, indicating generally narrower skulls (Fig. 4A), with extreme examples of this morphology apparent in the genera *Apostolepis*, *Phalotris*, and *Elapomorphus*. *Heterodon* and *Xenodon dorbignyi* are the exceptions to this general pattern (labeled in Fig. 4); in contrast to all other semi-fossorial species, they exhibit extremely wide skulls with a short snout. Aquatic snakes, representing species from three phylogenetically disparate clades, cluster very closely together in morphospace, indicating highly similar morphologies. No clear patterns for other habitat use groups emerge, although there is some overlap between cryptozoic and semi-fossorial taxa. Patterns in the non-trophic module morphospace with respect to diet are less clear; species specializing on mollusks and annelids seem to deviate in morphology from other species, however, the morphological correlates involved are not clear. Fish eating species also cluster to some extent (Fig. 4B, Fig. S2). A detailed account of the results we obtained from morphospace analyses for each of the remaining modules can be found in the supporting material (Additional file 4: Appendix S4).

When considering all modules, aquatic habitat use most often resulted in tight morphological clustering (as well as in deviation from other groups in the non-trophic and maxillary modules) and semi-fossorial habitat use most often resulted in deviations from other habitat use groups. In terms of diet, mollusk and annelid specialization most often resulted in a deviation from other morphologies.

For the majority of skull modules, PC1 and PC2 cumulatively explained more than 70% of shape variation (see discussion in Additional file 4: Appendix S4, Table S1), although this is at least partly a function of the number of landmarks considered per module (Table S1). For modules where this was not the case, such as the non-trophic module, variation in additional PC axes was qualitatively determined to either lack any clear trends across ecological groups or if trends were visible, they were redundant with trends exhibited by the first two PC axes. We therefore chose not to address variation in axes beyond PC2 in this manuscript.

### PGLS and PPLS analyses

Our PGLS analyses corroborated the trends visually identified in morphospace (Table 1). When considering multivariate shape data with procD.pgls, habitat use was a statistically significant predictor of shape in all 8 modules (Table 1). Primary diet was a statistically significant predictor of multivariate shape in the maxilla, mandible, and palatine modules, although in these modules it had a smaller effect size than habitat use (Table 1). Size significantly predicted shape in all modules except the ectopterygoid, but generally had a smaller effect size than habitat use (Table 1).

**Table 1:** Summary of statistics resulting from PGLS (procD.pgls – multivariate shape data and gls – univariate shape data) and PPLS analyses. Significant results (*P<*0.05) are highlighted in bold

**Module**	**Habitat Use (procD.pgls)**	**Primary Diet (procD.pgls)**	**Size (procD.pgls)**	**Habitat Use (PC1 – gls)**	**Primary Diet (PC1 – gls)**	**Size** **(PC1 – gls)**	**Habitat Use (PC2 – gls)**	**Primary Diet (PC2 – gls)**	**Size** **(PC2 – gls)**	**Diet (PPLS)**
**Non-Trophic**	**Z=6.2, *P=*0.001**	Z=0.64, *P=*0.264	**Z=5.56, *P=*0.001**	***P<*0.0001**	*P=* 0.415	***P=*0.002**	***P<*0.0001**	***P=*0.043**	*P=*0.979	**r-PLS=0.6, *P=*0.005**
**Maxilla**	**Z=6.27, *P=*0.001**	**Z=2.95, *P=*0.001**	**Z=2.1, *P=*0.022**	***P<*0.0001**	***P=*0.001**	*P=*0.87	***P=*0.036**	***P=*0.015**	*P=*0.086	**r-PLS=0.52, *P=*0.024**
**Ectopterygoid**	**Z=4.38, *P=*0.001**	Z=0.86, *P=*0.196	Z=-0.27, *P=*0.602	***P<*0.0001**	*P=*0.42	*P=*0.881	***P<*0.0001**	***P=*0.022**	*P=*0.206	r-PLS=0.37, *P=*0.138
**Supratemporal**	**Z=4.05, *P=*0.002**	Z=-0.72, *P=*0.762	**Z=1.79, *P=*0.039**	***P<*0.0001**	*P=*0.86	*P=*0.327	***P<*0.0001**	*P=*0.328	*P=*0.183	r-PLS=0.35, *P=*0.247
**Quadrate**	**Z=3.24, *P=*0.004**	Z=0.72, *P=*0.246	**Z=4.68, *P=*0.001**	*P=*0.067	*P=*0.16	***P<*0.0001**	***P<*0.0001**	*P=*0.426	***P=*0.017**	**r-PLS=0.45, *P=*0.048**
**Mandible**	**Z=4.19, *P=*0.001**	**Z=1.75, *P=*0.045**	**Z=3.54, *P=*0.001**	***P<*0.0001**	*P=*0.215	***P=*0.001**	***P=*0.012**	*P=*0.245	***P=*0.039**	**r-PLS=0.69, *P=*0.001**
**Pterygoid**	**Z=4.33, *P=*0.001**	Z=1.24, *P=*0.107	**Z=2.06, *P=*0.018**	***P=*0.001**	***P=*0.02**	*P=*0.411	***P<*0.0001**	*P=*0.729	*P=*0.094	**r-PLS=0.49, *P=*0.01**
**Palatine**	**Z=6.5, *P=*0.001**	**Z=1.84, *P=*0.032**	**Z=2.57, *P=*0.007**	*P=*0.08	*P=*0.053	*P=*0.081	***P<*0.0001**	***P<*0.0001**	*P=*0.005	**r-PLS=0.49, *P=*0.012**

Analyses of univariate shape data with the gls implementation of PGLS generated similar results when compared with multivariate shape analyses. Habitat use significantly predicted shape in all but the quadrate and palatine module when considering PC1 shape data and in all modules when specifying PC2 as the response variable. Primary diet significantly predicted shape (PC1) in the maxilla and pterygoid. For PC2, primary diet was a significant predictor of shape in the non-trophic module, maxilla, ectopterygoid, and palatine. Size significantly predicted shape in the non-trophic module, quadrate, and mandible for PC1 and the quadrate and mandible in PC2 shape data.

The phylogenetic partial least squares analysis using diet proportion data was largely consistent with the results obtained in the PGLS analyses. For the non-trophic module (r-PLS=0.6, *P=*0.01), maxilla (r-PLS=0.52, *P=*0.02), quadrate (r-PLS=0.45, *P=*0.048), mandible (r-PLS=0.69, *P=*0.001), pterygoid (r-PLS=0.49, *P=*0.01), and palatine (r-PLS=0.49, *P=*0.01), shape significantly covaried with diet. Consistent with most PGLS analyses, covariance of shape with diet in the ectopteryoid (r-PLS=0.37, *P=*0.14) and supratemporal module (r-PLS=0.35, *P=*0.25), were non-significant.

### Robustness of PGLS results

We assessed the robustness of the results obtained from the PGLS analyses through the application of a combined approach utilizing both real and simulated data. We compared PGLS analyses using the true grouping configuration (i.e. shape data associated with true corresponding ecological data) to those from datasets with categorical groupings randomly permuted across the tips. Note that this procedure both (1) removes any associations between shape and ecology present in the true data, but also (2) removes any phylogenetic signal that may be present in the ecological data. We also tested the effect of sample size (number of species) on the robustness of the results by repeating this test using varying sized subsets of the original dataset. 1000 repetitions of this simple permutation test were run for each of 25, 50, 75, 100, and 125 species subsets of the non-trophic module shape data using both procD.pgls and gls functions. We incorporated the same model as in our main PGLS results for the dataset, including size as a covariate.

**Fig. 5:**
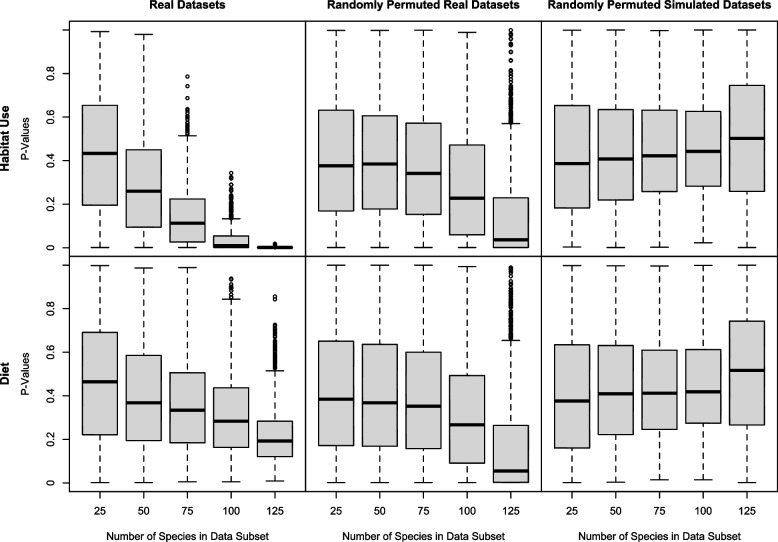
P-values from procD.pgls (geomorph) analyses of real (non-trophic module) shape datasets with true corresponding ecological data (left), those from analyses of real (non-trophic module) shape datasets paired with randomly permuted ecological data (middle), and those from analyses of BM-simulated shape data paired with randomly permuted ecological data (right). *P*-values are lower than expected for randomly permuted real datasets, an issue exacerbated with increased sample size. Retaining everything constant but simulating the shape data under BM produces expected *p*-values

For randomly permuted datasets, we find that p-values are skewed towards zero for both the habitat use and diet variable (Fig. 5 – center, Fig. S5 – center), thus indicating an increased Type I error rate for our analyses. This trend seems to become exacerbated as the data subset size increases. Note, however, that for the habitat variable, which significantly explained shape in the original analysis of the non-trophic module with procD.pgls (*P=*0.001), *p*-values across all real dataset subsets (Fig. 5 – top left, Fig. S5 – top left) are substantially lower than those with randomly permuted ecological groupings (Fig. 5 – top center, Fig. S5 – top center).

To determine whether the elevated Type I error rates observed under permutations were a property of the underlying PGLS framework or of our data, we conducted a third test using simulated data. First, we estimated the covariance matrix for the real non-trophic module shape data using the ratematrix function in the R package “geiger v2.0.10” [67, 68]. We then used the sim.char function in the same R package to create a simulated dataset of *n=*148 “species”, containing a multivariate response variable with *n=*116 variables (equal to the number of principal components of the non-trophic module). We simulated this multivariate response variable under Brownian motion on the same phylogeny used throughout this study, utilizing the covariance structure of the true shape data as estimated previously. We paired this simulated shape dataset with the true ecological data, size data, and phylogeny and repeated the random permutation robustness test as before.

Our results show that when retaining all factors constant (ecological data, size data, phylogeny, shape covariance structure) but simulating the response variable under multivariate BM, PGLS analyses on the datasets behave precisely as expected, with a median *p*-value of ~0.5 recovered with both functions and across all data subset sizes for both the habitat use and diet variable (Fig. 5 – right, Fig. S5 – right). These results suggest that PGLS may be sensitive to non-Brownian evolution of the underlying morphological traits. It is possible that many complex morphological traits that have been assessed in a PGLS framework show similar departures from underlying assumptions – namely, Brownian motion – such that the resulting distribution of *p*-values is unreliable. Robustness tests similar to those performed here are rarely, if ever, included in PGLS analyses to assess relationships between ecological predictors and multivariate morphological response variables. We thus recommend that researchers utilizing PGLS implementations for complex shape data consider the potential sensitivity of these methods to deviations from null assumptions.

## Discussion

Recent phylogenetic evidence [12, 69] suggests that dipsadines originated in the Old World before dispersing to the New World relatively early in their evolutionary history, subsequently diversifying into the tremendous diversity of species seen today. The exceptional diversity of trophic ecologies and foraging modalities within the dipsadines suggests that the diversification of this group may have been facilitated by ecological opportunity [1, 3, 70]. It is possible that South and Central America were, at the time of dipsadine colonization, relatively depauperate in highly-derived caenophidian snakes with evolutionarily versatile prey subjugation and processing phenotypes [10, 60], thus setting the stage for the rapid evolution of novel trophic ecologies observed in the group [71]. The apparently rapid lineage diversification in the dipsadines is also coupled with fast rates of trophic diversification and expansion of trophic space [71], further supporting the idea that the overall tempo and mode of this radiation reflects a response to ecological opportunity.

Nonetheless, it is overly simplistic to assume that dipsadines radiated in an ecological vacuum resulting from the simple absence of other snake lineages. Several major clades of "advanced" snakes were likely contemporaneous with dipsadines as they radiated [72], including colubrines [10], elapids, and viperids. Although the elapids in South America show substantially less diversity in morphology, diet, and habitat use than the dipsadines, related lineages of elapid snakes have undergone dramatic radiations in body form and ecology in other regions, most notably Australia [61, 73]. Hence, a key outstanding question in the assembly of New World reptile communities is whether dipsadine snakes radiated in response to extrinsic ecological opportunity, or whether they evolved novel phenotypes that facilitated subsequent ecological and lineage diversification even in the presence of potential competitor lineages (e.g., colubrine snakes; [10]). Several dipsadine lineages have acquired striking morphological [74] and histochemical [75, 76] adaptations that enable them to feed on unusual prey that are rarely consumed by other snake lineages, consistent with the hypothesis that the dipsadine bauplann is characterized by greater evolutionary versatility [77] than many other snake lineages.

We found that habitat and diet were significant predictors of shape in many dipsadine skull modules, across both multivariate and univariate shape data. Despite the statistical anomalies discussed in the above section, the *p*-values associated with ecological variables that significantly explained shape data in our analyses were substantially lower than with randomized data for the same modules/variables (Fig. 5, Fig. S5). Thus, our results suggest that ecological factors contribute to overall variation in skull shape across the dipsadine megaradiation. This is not to say that ecology is necessarily the *most* important factor in explaining variation in skull shape in dipsadines. Size was a significant predictor of shape across many modules, although it generally had a weaker signal than habitat use (Table 1). This is not an unexpected result, as many studies have shown that allometry often plays a role in explaining morphological disparity in skulls among vertebrates [8, 58, 78–81]. Although an in-depth discussion of allometric trends is outside the scope of this study, particularly given the dominance of ecological variables in explaining the data, we provide allometric regression plots in the supplemental results (Figs. S6, S7). We also cannot exclude the possibility that other untested factors such as pleiotropy, integration with other traits, or developmental constraints may play a more dominant role in explaining skull morphology than the variables tested here, and some studies have indeed shown that these factors can have a strong influence on skull evolution [6–8]. For instance, Bright and colleagues [8] found that in bird skulls, classically thought of as model examples of adaptation, the beak and braincase are highly integrated and developmentally constrained structures correlated with size, rather than independently evolving elements controlled by diet. Nonetheless, our results provide quantitative support for a correlation between ecology and morphology, long considered a central feature of adaptive radiation [1].

We showed that habitat use is a significant predictor of shape in a greater number of skull regions in comparison to diet and size, generally with higher effect sizes, indicating that habitat use may impose stronger selective pressures on the skull as a whole. Deepak et al. [82] recovered a similarly strong influence of habitat over diet in studies of natricine snake head morphology. In our study, the most distinct ecological groupings in morphospace included aquatic and semi-fossorial species, a finding that is consistent with Watanabe et al. [59] who recovered rampant convergence in skull shape within these ecological groups across squamates as a whole.

**Fig. 6:**
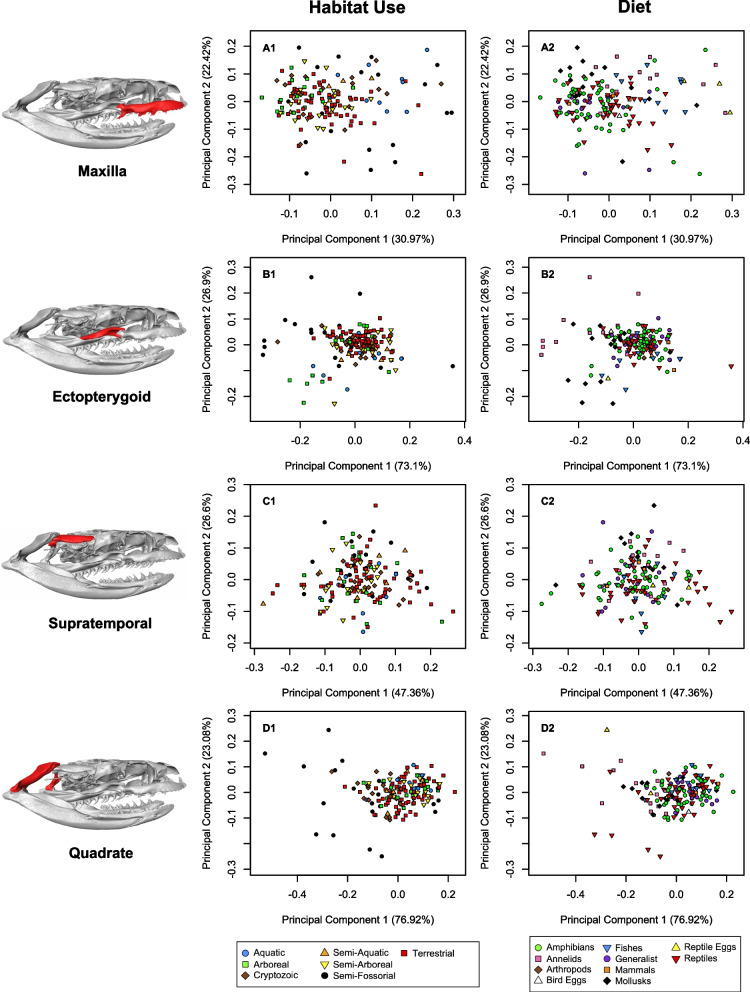
Principal components of shape (PC1, PC2) in four modules: maxilla (**A**), ectopterygoid (**B**), supratemporal (**C**), and quadrate (**D**) with respect to habitat use (1) and primary diet (2). Note deviation of aquatic snakes from the majority of other groups in the maxilla (A1). In the quadrate module, aquatic snakes group to the right, indicating longer quadrates, while semi-fossorial snakes generally group to the left, indicating short quadrates (D1, semi-fossorial outliers to right represent *Heterodon* and *Xenodon dornignyi*)

Savitzky [52] observed widespread convergence in aquatic/piscivorous snakes, noting that these species exhibit long quadrates in particular, which he hypothesized increased gape size for more rapid and effective consumption of difficult to handle fish prey. One study [54], corroborated this qualitative assessment with a morphometric study showing that among natricines, fish-eating species had relatively longer quadrates that appeared to significantly reduce prey-handling time. This finding was mirrored by Silva and colleagues [64] that found increased quadrate length in an aquatic coral snake relative to two terrestrial species. In our study, aquatic species also showed evidence of longer quadrates and tight clustering in morphological space with respect to quadrate shape (Fig. 6D1). We also found that aquatic snakes tightly clustered and tended toward an elongate skull in the analysis of the non-trophic module (Fig. 4A), a finding corroborated by several studies that speculated this to either function in increasing hydrodynamic efficiency or handling of fish prey [53, 55, 63, 83]. In cetaceans and crocodilians, aquatic clades of vertebrates that have both evolved varying degrees of elongation in the skull, McCurry and colleagues [84] found that extreme examples of elongation (i.e. gharials and river dolphins) were convergently evolved as a result of diet rather than specific aquatic habitat. As piscivory and adaptations for aquatic proclivities seem to be tied in vertebrates, disentangling these factors in dipsadine snakes will take further work and finer-scale ecological data.

The semi-fossorial group, with the exception of *Heterodon* and *Xenodon dorbignyi*, clustered fairly neatly away from other groups and towards a narrower skull in the non-trophic module analysis (Fig. 4A). This is consistent with Savitzky’s [52] finding that burrowers tend to have narrower, more rigid skulls, hypothesized to allow fossorial snakes to more effectively penetrate dense soils (see also [85] for detailed discussion on fossorial snake skull anatomy). Most semi-fossorial snakes also exhibit strong differentiation from other habitat use groups in their quadrate morphology – a finding corroborated by Palci et al. [66] in their study of squamate quadrate morphology, which again found that semi-fossorial snakes and lizards had significantly different quadrate morphology from terrestrial and aquatic species. With the exception of *Heterodon spp.*, we found that semi-fossorial snakes tended to have short, squat quadrates (the opposite of aquatic snakes), which may be tied to functional constraints associated with evolving elongate, but more compact skulls appropriate for soil penetration. Mediolaterally narrower skull shapes for burrowing versus non-burrowing species have also been recorded in fishes [86] and skinks [87], and dorsoventrally flattened skulls in salamanders [88]. Individuals with narrower skulls were shown to be faster burrowers in the caecilian *Schistometopum thomense* [89] and in amphisbaenians [90], although wider skull shapes are associated with higher absolute burrowing forces [91]. A more angular, wedge-shaped rostrum, exhibited by the semi-fossorial *Atractus*, *Heterodon*, and *Xenodon* (E, G, and H in Fig. 2, respectively), has been found in other fossorial snakes [92], fossorial lizards [93], burrowing fish [94], and forward-burrowing frogs [95]. In general, fossoriality seems to be a strong constraint on vertebrate skulls [96–100] often driving rampant homoplasy [45, 98, 101].

**Fig. 7:**
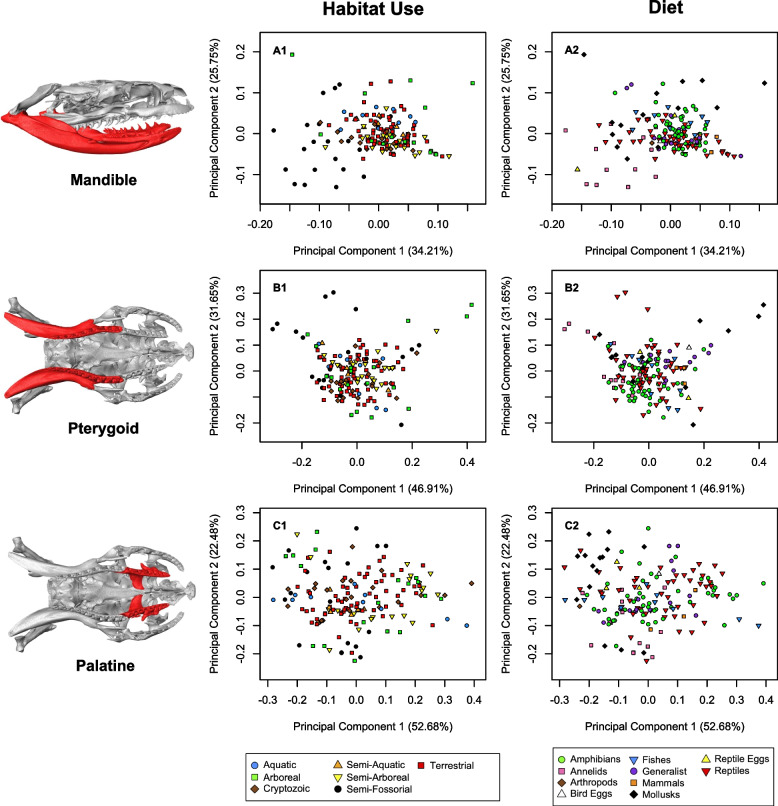
Principal components of shape (PC1, PC2) in three modules: mandible (**A**), pterygoid (**B**), and palatine (**C**) with respect to habitat use (1) and primary diet (2). Semi-fossorial species and mollusk and annelid specialists appear to be the only groups deviating visibly from others in certain modules

Our findings with respect to the diet variable corroborated some of the classic exemplars of trophic specialization in dipsadines. Mollusk and in particular snail specialists possess highly derived and unique traits that aid them in extracting and consuming their prey [52, 74, 76]. Our findings are consistent with this assessment, as in PC analyses of the ectopterygoid, maxilla, and palatine – all trophic components of the skull – mollusk specialists cluster in morphospace to some extent (Figs. 6, 7, S2, S4). Morphology associated with annelid prey specialization in snakes has not been discussed extensively to our knowledge. However, we suspect that the tendency we see for annelid specialists to deviate in morphospace in analyses of mandible and pterygoid shape (Figs. 7, S3, S4) relate to trophic constraints imposed by slippery, difficult to handle prey.

Our results suggest several instances of convergent evolution, evidenced both quantitatively in morphospace (Fig. 4) and upon inspection of the skulls themselves (Fig. 2). One of the most conspicuous examples of this phenomenon involves the semi-fossorial outliers: *Heterodon* and *Xenodon dorbignyi*. These two taxa represent phylogenetically and geographically disparate lineages that visibly converge on the same area of morphospace, both clustering, however, at the extreme opposite end of morphospace when compared to all other semi-fossorial species (Fig. 4A). Although both species are burrowers [102, 103], they probably spend most of their time above ground [104] and may use their expanded and prominent rostrum to unearth toad prey during the daytime [102]. This is a very different life history than most truly fossorial or semi-fossorial snakes and may account for their morphological distinctiveness relative to other purportedly fossorial/semi-fossorial snakes. Strikingly similar skulls with expanded and upturned rostra have also evolved in another diurnal toad forager, *Leioheterodon* [105, 106], a phylogenetically distant lineage from Madagascar. Having independently evolved in several phylogenetically and geographically distinct lineages, it is probable that this highly specialized morphotype has strong ecological correlates, despite the fact that our data were unable to detect them.

Unfortunately, we lack even basic natural history data for many snakes [107, 108] and it is probable that the coarse ecological state codings used here have masked complexity in the relationship between habitat, diet, and skull morphology. As several authors have noted [108–110], better-quality and finer-scale ecological data are badly needed to understand snake diversification more generally [71]. The paucity of ecological data for snakes results in part from their inherently cryptic nature and the difficulty in obtaining large sample sizes [111]. Their subsequently poor reputation as ecological study models has resulted in comparatively few snake-focused ecological publications [107, 108], but see [112]. A concerted effort to gather more and better ecological data for snakes is more than warranted, particularly when considering this taxon’s potential as a system for understanding large-scale biodiversity phenomena. Snakes comprise many overlooked but spectacular examples of adaptive radiation [61, 71], key innovation [48, 49, 60], evolutionary arms races [113–115], and Mullerian-Batesian mimicry rings [72, 116, 117]. As our understanding of snake natural history increases, we believe that they will come to represent a rich source of insight into macroevolutionary and ecological dynamics.

## Conclusions

With dipsadine snakes, habitat use and diet are strong predictors of skull shape. This finding is consistent with the idea that dipsadines have radiated in response to ecological opportunity, an idea that is further supported by the tempo and mode of trophic niche evolution within the group [71]. More evidence is required to definitively support the idea that the spectacular ecological and phenotypic diversity of the dipsadines is the result of adaptive radiation, however, the findings of this work serve to satisfy one of the requirements, namely, a correlation between ecology and morphology. We find that habitat ecology may play a more prominent overall role in constraining skull shape in dipsadines when compared to diet, although both significantly correlated with the shape of many regions of the skull. Fossorial and aquatic dipsadines separated from other habitat use groups most strongly; we argue that this is likely associated with the strong constraints imposed by the difficult mediums (soil and water, respectively) that these snakes tend to move through. Lastly, we identify an instance where our coarse ecological data were unable to illuminate the circumstances of a probable instance of convergence. As one of the largest continental vertebrate radiations, there is an acute need for comprehensive ecological, phenotypic, and phylogenetic information from dipsadines such that we might better understand both the evolutionary causes and macroecological consequences of their diversification.

## Methods

### Overview

We generated cranial images for 160 species of dipsadines using x-ray micro-computed tomography (hereafter, CT) scanning technology. We digitally constructed 3-dimensional surface models for each skull and quantified their shape using geometric morphometrics. We compiled ecological data from the primary literature and through a recently published predator-prey interaction database for snakes [112]. We used principal components analysis to quantify and visualize the variation in skull shape across the dataset, after removing the effects of rotation and minimizing size differences in the shape data. We then used phylogenetic generalized least-squares (PGLS) and phylogenetic partial least-squares (PPLS) to explore the contribution of ecological variables to the observed variation in skull shape in a phylogenetic framework, while accounting for potential allometric effects. Finally, we applied a set of permutation and simulation based analyses to explore the robustness of the relationships between ecology and cranial morphology.

### Morphological dataset

We collected 3D morphological images for 160 species of dipsadine snakes using micro-CT. We selected one representative specimen, free of skeletal damage, for each species (specimens examined in Additional file 1: Appendix S1). Specimens utilized were obtained primarily from the publicly available holdings of the University of Michigan Museum of Zoology – a full list of specimens used, with corresponding museum institution codes and catalog numbers, is available in the supplementary material (Additional file 1: Appendix S1). We selected only adult specimens, assessed by comparing material for the species, as juvenile snake skull morphology is significantly different from adult skull morphology in many cases [118–121] and specifically for dipsadines [122, 123], which could mislead an analysis of skull morphology across species. Many juvenile snakes exhibit classically paedomorphic features such as proportionally enlarged eyes and braincase and a larger head relative to their body size [118–123], and these traits often easily distinguish juveniles from adults when sufficient comparative material is available. We did not include species where adequate comparative material was not available and all specimens appeared to exhibit paedomorphic traits.

We obtained micro-CT scans primarily using a Nikon XT H225ST μCT machine, though several were imaged on a Scanco Medical µCT 100. Our scanning parameters varied depending on the specimen and facility, but ranged from 10-30 μm voxel size, 85-100 kV, 80-200 mA, 500-1600 projections, and 1-2 frame averaging. We reconstructed tomograms from raw radiograph projections on the Nikon XT H225ST machines using proprietary Nikon CT pro 3D software. We used the program Avizo to create 3-dimensional surface models on which landmarks could be placed.

**Fig. 8:**
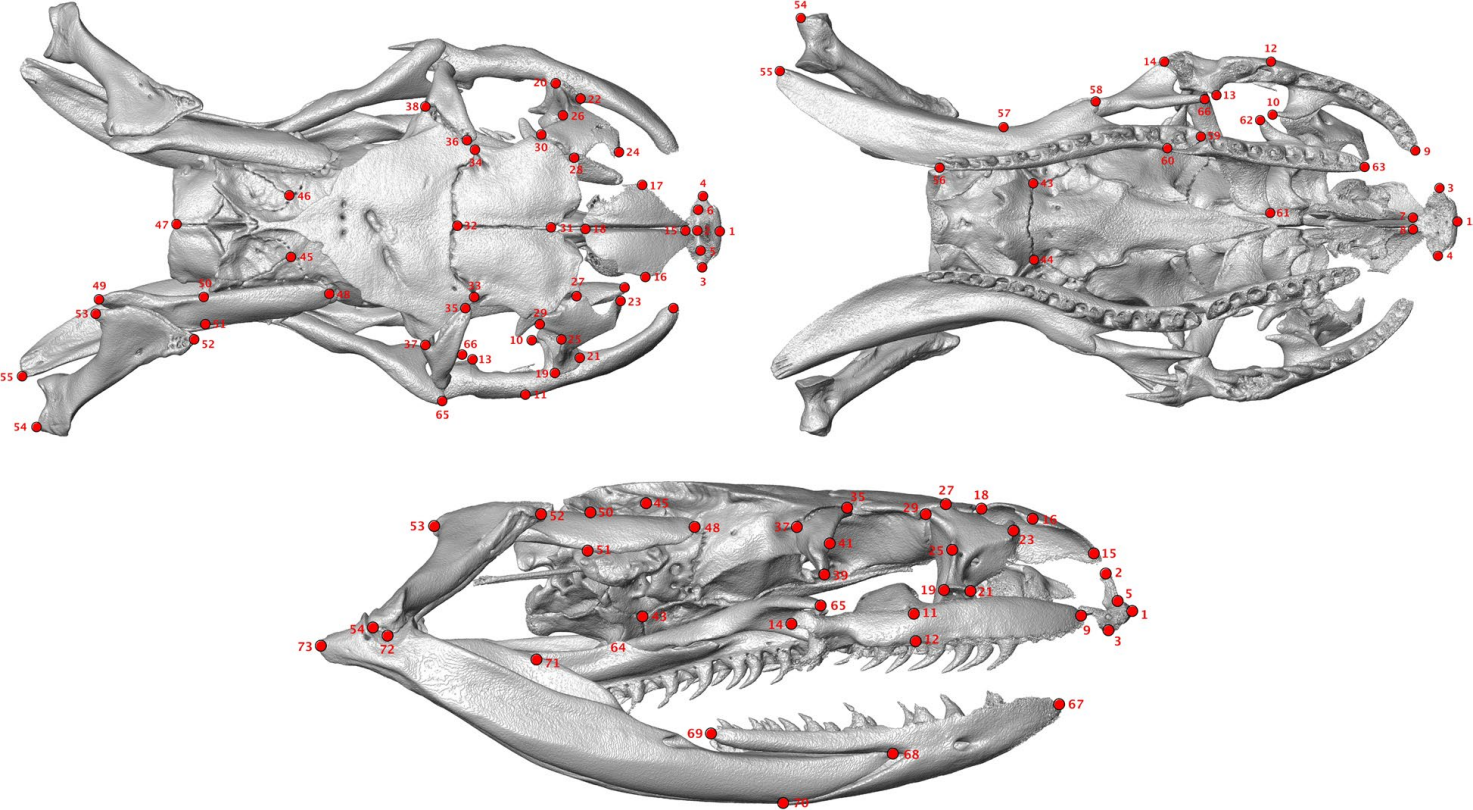
Locations for all 73 landmarks scored in this study. Note that symmetrical sides of cranium were landmarked; trophic structures that were independently analyzed were landmarked right side only

We chose landmark locations (*n=*73) (Fig. 8, Additional file 2: Appendix S2) based on easily identifiable and homologous locations on skull elements that were present and unambiguous across all taxa. We attempted to capture the maximal amount of observed morphological variation across species with our landmarking scheme. We placed landmarks on both sides of the cranium, as there is evidence that landmarking only single halves of bilaterally symmetrical structures can influence the accuracy of geometric morphometrics analyses [124]. Disjoint trophic structures that we analyzed separately were landmarked right side only. We landmarked surface models using Stratovan Checkpoint.

### Ecological data

We obtained habitat use data through general literature surveys as well as from personal experience in observing species in the field (Additional file 3: Appendix S3). We qualitatively assessed each of the 160 dipsadine species as being one of the following: semi-fossorial (partially subterranean dwelling), cryptozoic (highly cryptic, found below leaf litter, logs etc.), terrestrial, semi-arboreal, arboreal, semi-aquatic, and aquatic.

We compiled dietary data in two separate ways. For a smaller species subset (*n=*68), we assessed the data quantitatively in the form of proportion of each prey category consumed. For the entire dataset (*n=*160), we assessed diet categorically in the form of primary prey category, utilizing a combined quantitative and qualitative approach. We extracted quantitative dietary data from SquamataBase, a database consisting of predator-prey interactions for over 1,200 species of snakes [112]. Each of the records in this database represents a single observation of a prey item consumed by a snake. These records were either obtained from published literature or through dissections of museum specimens. These quantitative dietary data were available for 129 of the species in the dataset. Of these 129 species with quantitative dietary data, 68 species were represented by greater than 10 dietary records. We compiled the dietary data for this 68 species subset in the form of proportion prey consumed by each species in each of 10 categories: amphibians, reptiles, birds, mammals, fish, reptile eggs, bird eggs, annelids, and mollusks. Our diet-coding scheme closely follows prey taxonomy. For the entire 160 species dataset, we then assessed diet categorically. For species with quantitative diet data available, we assigned primary diet category based on which prey type accounted for over 50% of a given species diet. For species in which no one category made up more than 50% of the total prey composition, we assigned the category of “generalist”. For the remaining 31 species for which no quantitative diet data was available, we assessed primary diet category qualitatively based on the available literature. All ecological coding designations and corresponding references are available in Additional file 3: Appendix S3.

### Phylogenetic framework

Morphology across species will to some extent be correlated as a consequence of shared evolutionary history; species thus do not represent independent data points that can be directly compared [125–128]. We accounted for phylogenetic structure in the data through the use of comparative methods incorporating phylogenetic relationships, extended for use with geometric morphometric data [129–132]. The phylogeny used in all analyses was a recent time-calibrated molecular phylogeny for 1263 species of advanced caenophidian snakes, produced utilizing a supermatrix approach in a maximum likelihood framework [12]. From the full 160 species dataset, 148 species were present in the Zaher tree; consequently, we restricted statistical analyses to this 148 species subset but visualized morphospace for the full 160 taxon dataset.

### Analyses

We assessed variation in skull shape across the dataset after removing the effects of rotation and minimizing size differences in the landmark data. This was done by visualizing species positions in the morphospace defined by the first two principal components of the Procrustes-transformed landmark data. We then tested whether habitat use and diet composition were significant predictors of skull shape. We included size as an additional explanatory variable in our analyses, to account for any possible effects of allometry.

For analysis, we subset landmark coordinates for a total of 8 skull modules that we then analyzed in isolation: non-trophic elements (braincase, postorbitals, prefrontals, nasals and premaxilla), maxilla, ectopterygoid, supratemporal, quadrate, mandible, pterygoid, and palatine (Fig. 3). Because there are many kinetic elements in the snake skull that are capable of rotation and translation, a global analysis of overall skull shape would be confounded by the arbitrary position that these elements were preserved in. Various methods are available for removing the effects of arbitrary rotation and translation in articulated 3D structures [133–135]. However, the large number of extremely loose articulations in the snake skull, combined with their mobility on multiple axes, makes implementing some of these methods (i.e. [133]) inhibitive and ineffective for the 3-dimensional snake skull [136]. Although other methods (e.g. [134, 135]) can accommodate complex, linking chains like those that occur in the snake cranium, we nonetheless chose to analyze the most mobile elements individually. This both removed the effect of arbitrary element rotation as a result of kinesis and allowed us to more easily detect fine-scale variation in each element independently.

We conducted analyses in R 4.2.2, utilizing the packages “geomorph v.4.0.5” [137, 138], “RRPP v1.3.1” [139, 140], “ape v.5.7” [141], “nlme v3.1-162” [142, 143], “phytools v1.5-1” [144], and “car v3.1.1” [145]. We also created certain visualizations using functions from “diversitree v.0.9-16” [146]. We provide all relevant data (landmarks, ecological data etc.) along with an annotated R script containing code used for all procedures described in this paper in the Dryad Digital Repository, under the https://doi.org/10.5061/dryad.6q573n63n.

We aligned landmarks and minimized size differences for each skull module across all 160 species using Generalized Procrustes Analysis [147, 148]. We conducted principal components analyses for all aligned modules separately, plotting PC scores for principal components 1 and 2 and differentially coloring points based on habitat use and primary diet to visualize variance in skull shape with respect to these ecological variables. We obtained the mean shape for the non-trophic skull module across all species and warped it using the thin-plate spline approach [149] to represent the shape extremes along the plotted PC axes. This essentially allows for the interpretation of shape variation tracked by each principal component axis. We also conducted principal components analyses for the subset of species with proportional diet data (*n=*68) across all aligned skull modules. We then plotted PC scores for PC 1 and 2 as pie chart points in morphospace representing each species’ diet composition (figures available in Additional file 4: Appendix S4).

### Ecological predictors of cranial evolution

We tested the effects of habitat use and primary diet on skull shape using phylogenetic generalized least squares (PGLS) with permutation. As size (allometry) has been shown to be a significant contributing factor to shape in many groups [58, 78–81], we designated size as an additional explanatory variable. We used log of centroid size (the square root of the sum of squared distances of the landmarks to their centroid, obtained from general Procrustes analyses), as a proxy for skull size. We implemented this PGLS analysis using the function “procD.pgls” in geomorph [129, 131, 132]. We used a single model, with three independent variables (habitat use, primary diet, and size) and one dependent variable (skull shape). Modeling independent variables together allowed us to compare the predictive strength of these variables on the shape of each skull module. Because R^2^ values are difficult to interpret for multivariate data [Adams, pers. comm.], we computed and compared the effect size as a metric for comparing the strength of signals. As procD.pgls can accommodate 3-dimensional, aligned landmark coordinates, we used these to represent skull shape. We calculated covariance across species as a result of phylogenetic relationships under a Brownian motion model of evolution.

We applied a second PGLS implementation using “gls” in the nlme package, “Anova” in the car package, and simplified shape data, in order to provide an additional layer of robustness to the results. Whereas with procD.pgls we utilized the unaltered high-dimensional shape dataset, here we used PC scores for principal components 1 and 2 of skull shape as the shape variables in two separate analyses. In order to account for phylogenetic structure, we specified an evolutionary covariance matrix under Brownian motion, using the function “corBrownian” from the ape package. We utilized the same model structure and variables as with procD.pgls.

We assessed the correlation between diet in the form of proportional data and skull shape using two-block partial least squares in a phylogenetic context (PPLS). This method assesses the degree to which two sets of variables covary in a phylogenetic context, assessing significance with a permutation test [150, 151]. Thus, it is not a test that assesses the effect of independent variables on a dependent variable as in PGLS, but rather the degree to which the two sets of variables are correlated, with no assumption of directionality [152]. We implemented PPLS using the function “phylo.integration” in geomorph [130, 132].

### Supplementary Information

**Additional file 1.** Specimens used in this study, with corresponding museum voucher numbers.

**Additional file 2.** A list and description of all landmark positions utilized in this study.

**Additional file 3.** Codings for habitat and major diet ecology for all species analyzed in this study, including literature references used to infer these data.

**Additional file 4: Table S1.** Variance explained by the first ten PC axes across skull modules, for the full 160 species dataset. Note that variance explained for most skull modules, even those with high numbers of landmarks, drops significantly after PC4. **Fig. S1.** A snapshot of the ecological diversity of the Dipsadine mega-radiation with taxon labels. **Fig. S2.** Principal components of shape (PC1, PC2) in three modules: non-trophic (A), maxilla (B), ectopterygoid (C), with respect to proportional diet composition, illustrated as pie-chart points. **Fig. S3.** Principal components of shape (PC1, PC2) in three modules: supratemporal (A), quadrate (B), mandible (C), with respect to proportional diet composition, illustrated as pie-chart points. **Fig. S4.** Principal components of shape (PC1, PC2) in two modules: pterygoid (top) and palatine (bottom) with respect to proportional diet composition, illustrated as pie-chart points. **Fig. S5.** P-values from gls (nlme) analyses of real datasets with true corresponding ecological data (left), those from analyses of real datasets paired with randomly permuted ecological data (middle), and those from analyses of BM-simulated shape data paired with randomly permuted ecological data (right). P-values are lower than expected for randomly permuted real datasets, an issue exacerbated with increased sample size. Retaining everything constant but simulating the shape data under BM produces expected p-values. **Fig. S6.** Allometry plots (regression of size against shape) in four modules: non-trophic (A), maxilla (B), ectopterygoid (C), and supratemporal (D) with respect to habitat use (1) and primary diet (2). Allometry appears to be the strongest in the non-trophic module, with looser allometric relationships visible in the maxilla and supratemporal. A rough pattern of isometry is visible in the ectopterygoid. **Fig. S7** . Allometry plots (regression of size against shape) in four modules: quadrate (A), mandible (B), pterygoid (C), and palatine (D) with respect to habitat use (1) and primary diet (2). Allometry appears to be the strongest in the quadrate and mandible, with a looser pattern between size and shape in the pterygoid and palatine.

## Data Availability

We have included a zipped archive of all data files and scripts in our submission that we would like to make available during the review process. All data and associated scripts for analysis is also archived in the Dryad Data Repository, under the following https://doi.org/10.5061/dryad.6q573n63n.

## Abbreviations

CT: Computed tomography
PGLS: Phylogenetic generalized least-squares
PPLS: Phylogenetic partial least-squares
