# Supplementary material for: Ecological correlates of cranial evolution in the megaradiation of dipsadine snakes
Source: BMC Ecol Evol. 2023 Sep 8;23:48. doi: 10.1186/s12862-023-02157-3 (PMC10485986; doi:10.1186/s12862-023-02157-3)
Supplement: Supplementary file 4 — Additional file 4: Table S1. Variance explained by the first ten PC axes across skull modules, for the full 160 species dataset. Note that variance explained for most skull modules, even those with high numbers of landmarks, drops significantly after PC4. Fig. S1. A snapshot of the ecological diversity of the Dipsadine mega-radiation with taxon labels. Fig. S2. Principal components of shape (PC1, PC2) in three modules: non-trophic (A), maxilla (B), ectopterygoid (C), with respect to proportional diet composition, illustrated as pie-chart points. Fig. S3. Principal components of shape (PC1, PC2) in three modules: supratemporal (A), quadrate (B), mandible (C), with respect to proportional diet composition, illustrated as pie-chart points. Fig. S4. Principal components of shape (PC1, PC2) in two modules: pterygoid (top) and palatine (bottom) with respect to proportional diet composition, illustrated as pie-chart points. Fig. S5. P-values from gls (nlme) analyses of real datasets with true corresponding ecological data (left), those from analyses of real datasets paired with randomly permuted ecological data (middle), and those from analyses of BM-simulated shape data paired with randomly permuted ecological data (right). P-values are lower than expected for randomly permuted real datasets, an issue exacerbated with increased sample size. Retaining everything constant but simulating the shape data under BM produces expected p-values. Fig. S6. Allometry plots (regression of size against shape) in four modules: non-trophic (A), maxilla (B), ectopterygoid (C), and supratemporal (D) with respect to habitat use (1) and primary diet (2). Allometry appears to be the strongest in the non-trophic module, with looser allometric relationships visible in the maxilla and supratemporal. A rough pattern of isometry is visible in the ectopterygoid. Fig. S7 . Allometry plots (regression of size against shape) in four modules: quadrate (A), mandible (B), pterygoid (C), and palatine (D) w [file 12862_2023_2157_MOESM4_ESM.pdf]

## APPENDIX S4 – SUPPLEMENTARY RESULTS AND FIGURES

### PC Axes

In general, proportion of variance explained by the first few PC axes across modules scaled with number of landmarks in that module (Table S1). In cases of modules with very few landmarks, such as the ectopterygoid and quadrate, most or all of the variation was explained by the first two PC axes. In the maxilla, the first two principal components accounted for 53.4% and 58.5% in the full dataset and 68 species subset with proportional diet data, respectively (Table S1, Fig. 6, Fig. S2). In the ectopterygoid, PC1 and PC2 accounted for 100% of shape variation in both datasets, with PC1 accounting for a majority of the variation (Table S1, Fig. 6, Fig. S2). Increasingly negative PC1 values represent long, thin ectopterygoids while positive values represent increasingly short and wide ectopterygoids. The ectopterygoid landmark subset was restricted to 3 landmarks, which is why shape variation could be fully described by only 2 axes. Although there are additional PC axes technically present, the full variation of this shape (essentially a triangle) can be described by two parameters, therefore accounting for the fact that the remainder of the axes explain 0% of the variation. In the supratemporal, PC1 and PC2 accounted for 74% and 78.2% in the full dataset and 68 species subset, respectively. Most variation was accounted for by the first principal component (Table S1, Fig. 6, Fig. S3). In the quadrate module, the cumulative variation explained by the first two PC axes in both cases was 100%, for the same reason as the case of the quadrate. Most variation in this case was also explained by the first PC axis (Table S1, Fig. 6, Fig. S3). Negative PC1 values represented short and wide quadrates while positive values represented long, thin quadrates. In the mandible, the first two PC axes describe 60% and 60.3% of the shape variation in the full dataset and 68 species subset, respectively. Variation here is more equally distributed between PC1 and PC2 (Table S1, Fig. 7, Fig. S3). In the case of the pterygoid, the first two PC axes account for 78.6% of the variation in the full dataset and 79.4% of the variation in the 68 species subset. Most of the variation is described by the first principal component (Table S1, Fig. 7, Fig. S4). Shape variation in the palatine module is mostly described by the first principle component, with 75.2% and 79% of the variation accounted for by the first two principal components in the full dataset and 68 species subset, respectively (Table S1, Fig. 7, Fig. S4). For the 68 species subset with respect to the non-trophic component, PC1 and PC2 explained 53.3% of the variation.

| Module | Landmarks | PC1 | PC2 | PC3 | PC4 | PC5 | PC6 | PC7 | PC8 | PC9 | PC10 |
|---|---|---|---|---|---|---|---|---|---|---|---|
| **Non-Trophic** | 41 | 0.27 | 0.19 | 0.12 | 0.06 | 0.05 | 0.05 | 0.03 | 0.03 | 0.02 | 0.02 |
| **Maxilla** | 6 | 0.31 | 0.22 | 0.15 | 0.12 | 0.05 | 0.05 | 0.03 | 0.03 | 0.02 | 0.01 |
| **Ectopterygoid** | 3 | 0.73 | 0.27 | N/A | N/A | N/A | N/A | N/A | N/A | N/A | N/A |
| **Supratemporal** | 4 | 0.47 | 0.27 | 0.15 | 0.10 | 0.01 | N/A | N/A | N/A | N/A | N/A |
| **Quadrate** | 3 | 0.77 | 0.23 | N/A | N/A | N/A | N/A | N/A | N/A | N/A | N/A |
| **Mandible** | 7 | 0.34 | 0.26 | 0.15 | 0.10 | 0.05 | 0.04 | 0.02 | 0.01 | 0.01 | 0.01 |
| **Pterygoid** | 5 | 0.47 | 0.32 | 0.08 | 0.06 | 0.03 | 0.02 | 0.01 | 0.01 | N/A | N/A |
| **Palatine** | 4 | 0.53 | 0.22 | 0.13 | 0.07 | 0.04 | N/A | N/A | N/A | N/A | N/A |

**Table S1. Variance explained by the first ten PC axes across skull modules, for the full 160 species dataset.** Note that variance explained for most skull modules, even those with high numbers of landmarks, drops significantly after PC4.

**Morphospace**

In the maxillary module, there do not seem to be any obvious groupings of morphologies based on habitat, although aquatic snakes tend to group more towards positive PC values than other groups and semi-fossorial snakes tend to inhabit the peripheries of the morphospace. With respect to diet, most amphibian eaters and mollusk eaters cluster quite closely, although this is within the variation of other groups (Fig. 6, Fig. S2).

Analysis of the ectopterygoid module revealed that semi-fossorial snakes inhabit the periphery of the morphospace, but do not form a tight cluster. With respect to diet, mollusk and some annelid specialists tend towards having longer, thinner ectopterygoids (Fig. 6, Fig. S2).

In the supratemporal, mollusk specialists appear to cluster to some extent towards the positive extreme of PC2 (Fig. 6, Fig. S3). No other trends are immediately noticeable with respect to habitat or diet groupings in this module.

With respect to the quadrate, aquatic snakes seem to show much less diversity in morphology than other groups and are clustered towards the positive end, exhibiting relatively longer quadrates. Semi-fossorial snakes (except Heterodon, Xenodon dorbignyi outliers) and cryptozoic snakes tend to deviate in the opposite direction, exhibiting shorter, stouter quadrates. No clear patterns are visible with respect to diet; piscivores seem to cluster closely to some extent (Fig. 6, Fig. S3).

In the mandible module, semi-fossorial snakes cluster widely, but consistently, toward minimum PC1 values. As with many other modules, aquatic snakes cluster much more tightly than other groups. For diet, annelid specialists cluster away from other groups, toward minimum PC1 values (Fig. 7, Fig. S3).

In the pterygoid, no clear patterns with respect to habitat are visible; diet relations are also ambiguous although annelid specialists tend to cluster towards minimum PC1 values (Fig. 7, Fig. S4).

Within the palatine module, plotting with respect to habitat yields an ambiguous result. With respect to diet, however, all but one mollusk specialist clusters at minimum PC1 and PC2 values, away from other morphologies (Fig. 7, Fig. S4).

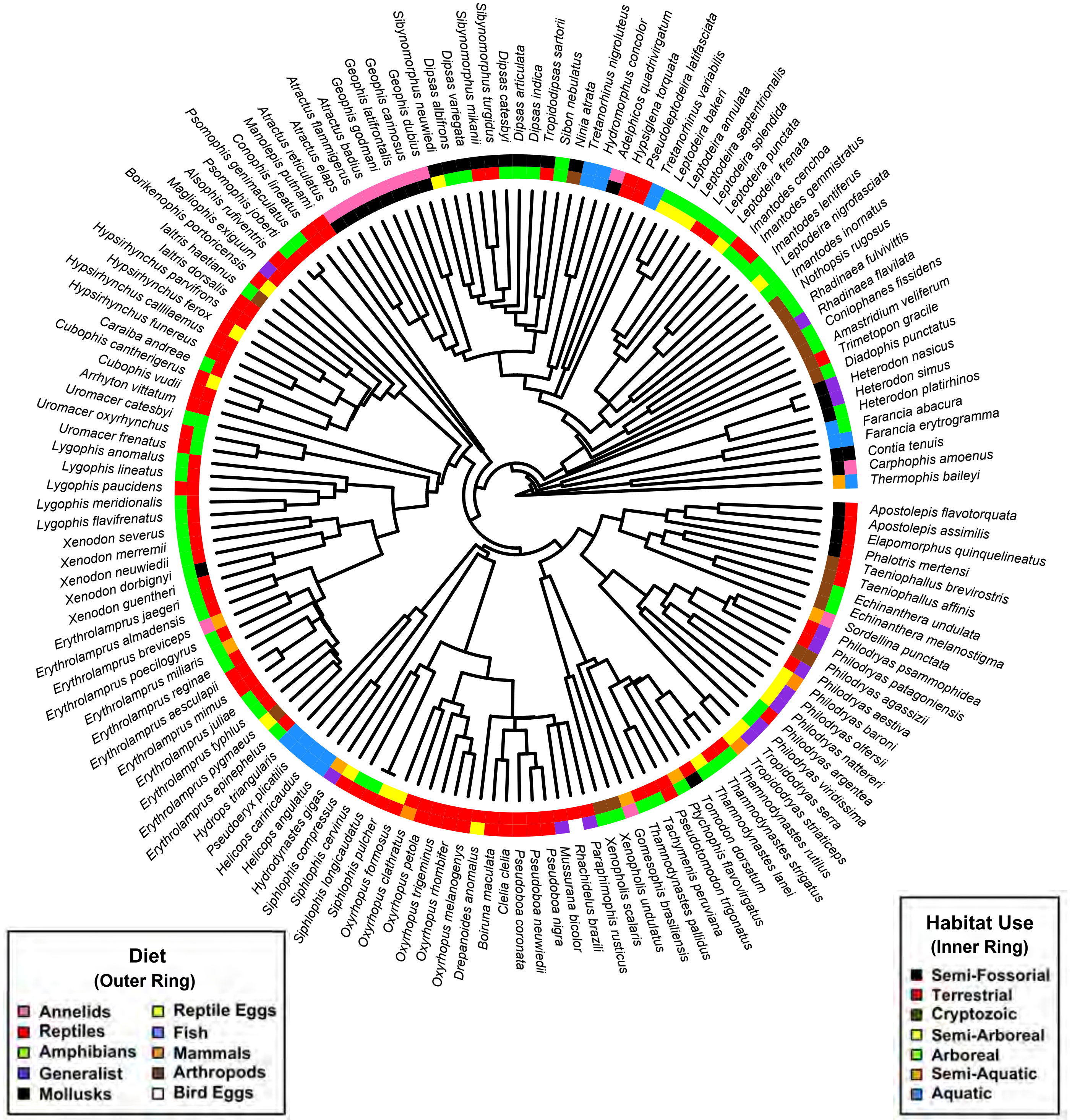


**Fig. S1. A snapshot of the ecological diversity of the Dipsadine mega-radiation with taxon labels.**

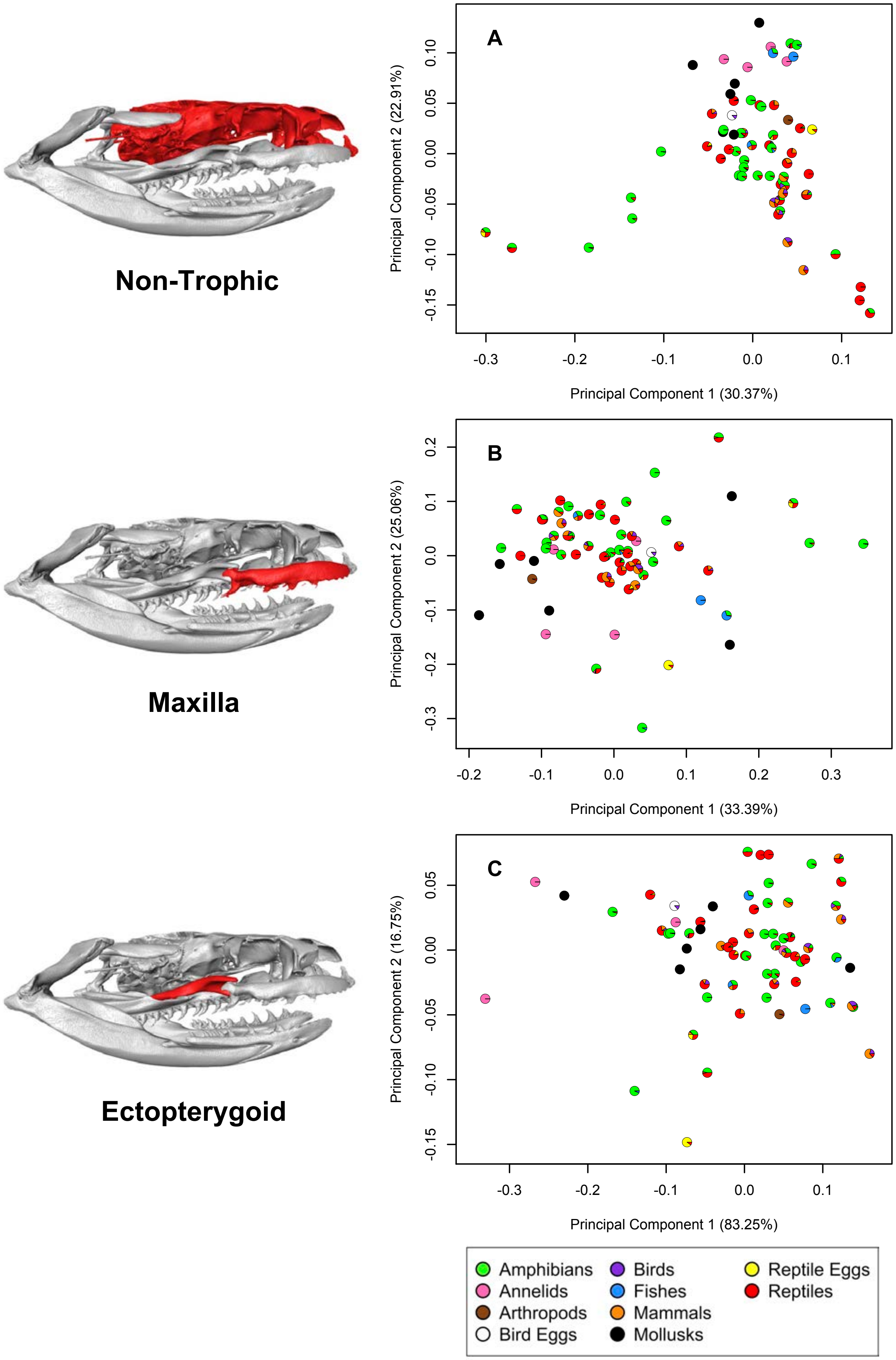


**Fig. S2. Principal components of shape (PC1, PC2) in three modules: non-trophic (A), maxilla (B), ectopterygoid (C), with respect to proportional diet composition, illustrated as pie-chart points.**

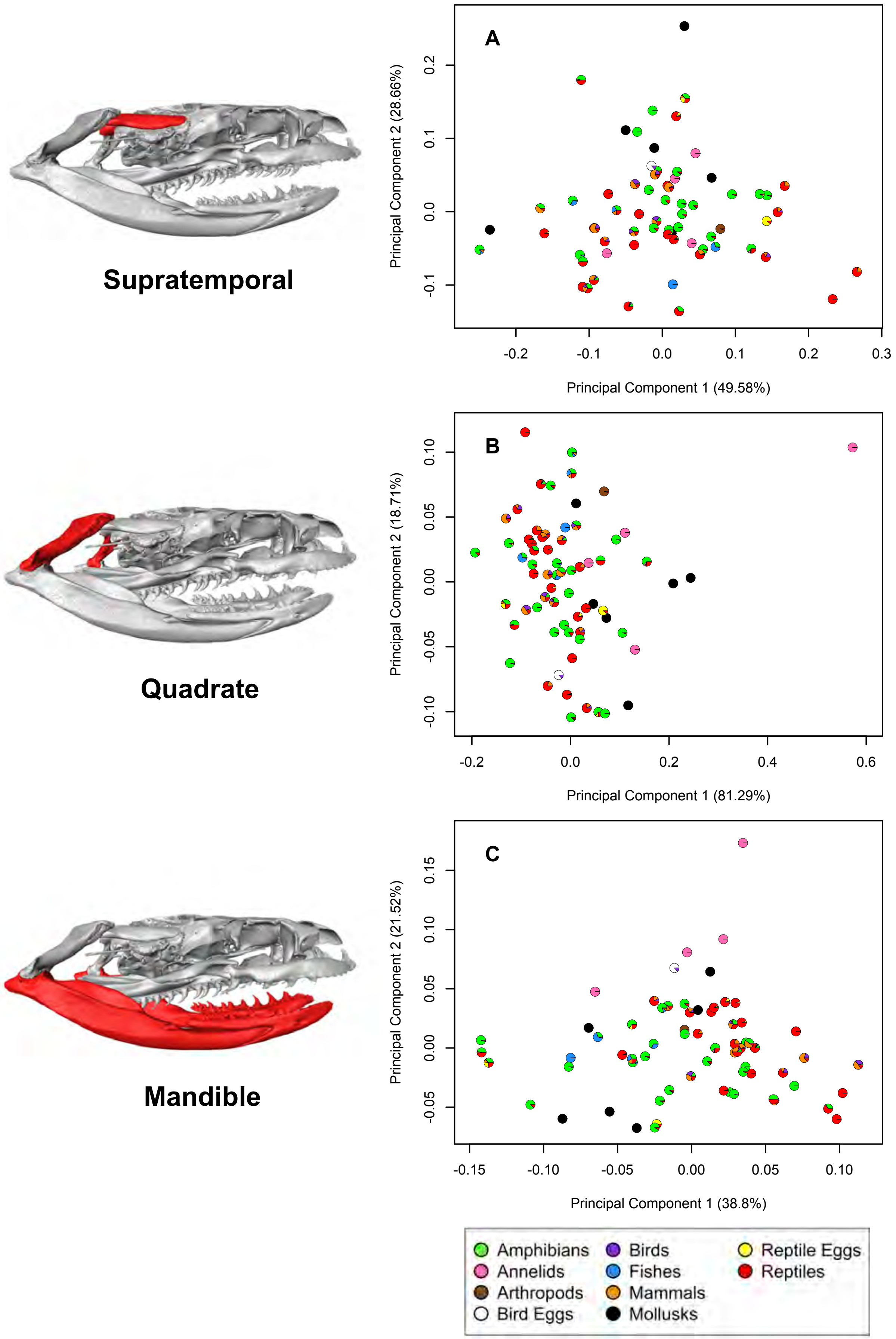


**Fig. S3. Principal components of shape (PC1, PC2) in three modules: supratemporal (A), quadrate (B), mandible (C), with respect to proportional diet composition, illustrated as pie-chart points.**

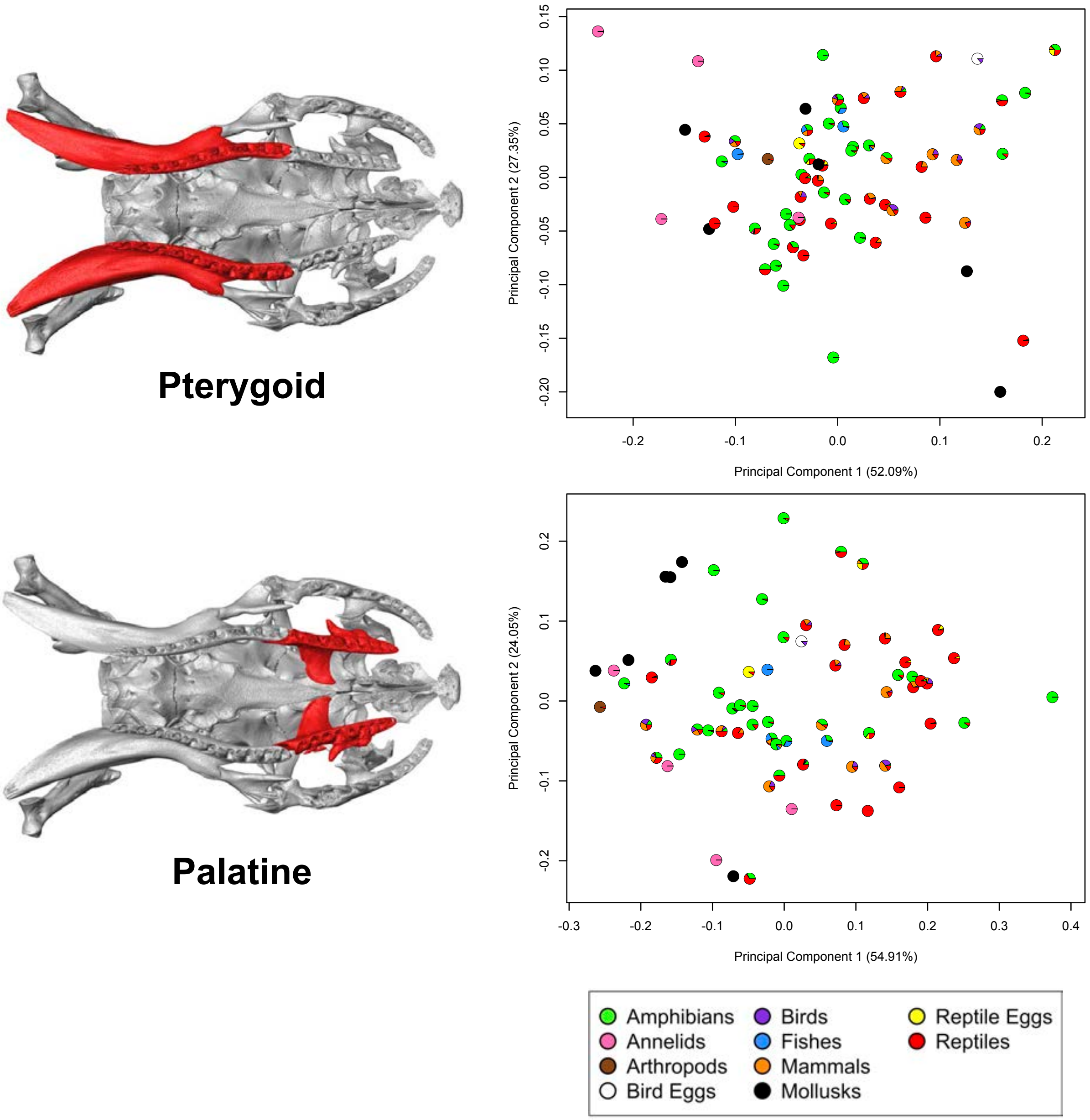


**Fig. S4. Principal components of shape (PC1, PC2) in two modules: pterygoid (top) and palatine (bottom) with respect to proportional diet composition, illustrated as pie-chart points.**

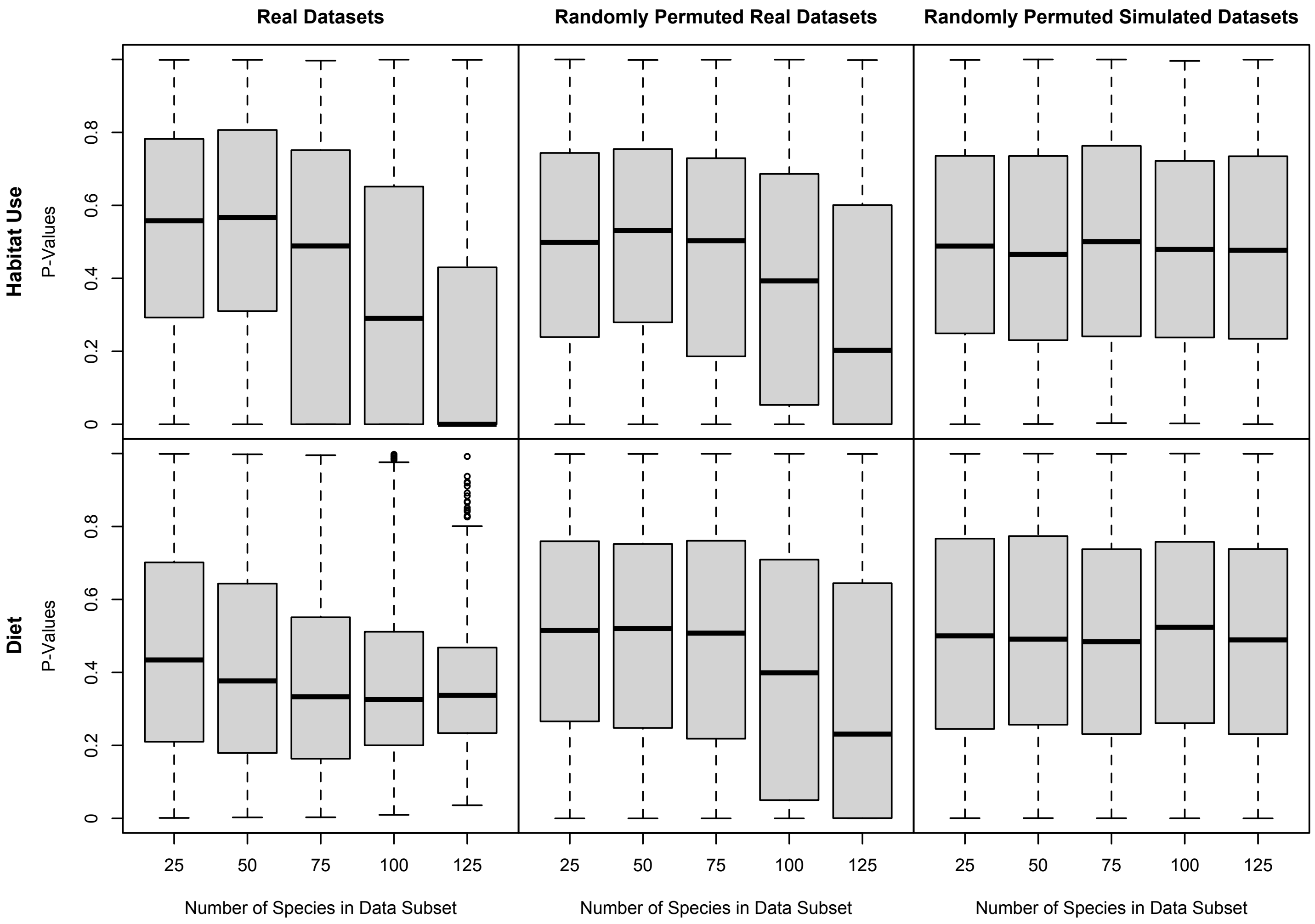


**Fig. S5. P-values from gls (nlme) analyses of real datasets with true corresponding ecological data (left), those from analyses of real datasets paired with randomly permuted ecological data (middle), and those from analyses of BM-simulated shape data paired with randomly permuted ecological data (right).** P-values are lower than expected for randomly permuted real datasets, an issue exacerbated with increased sample size. Retaining everything constant but simulating the shape data under BM produces expected p-values.

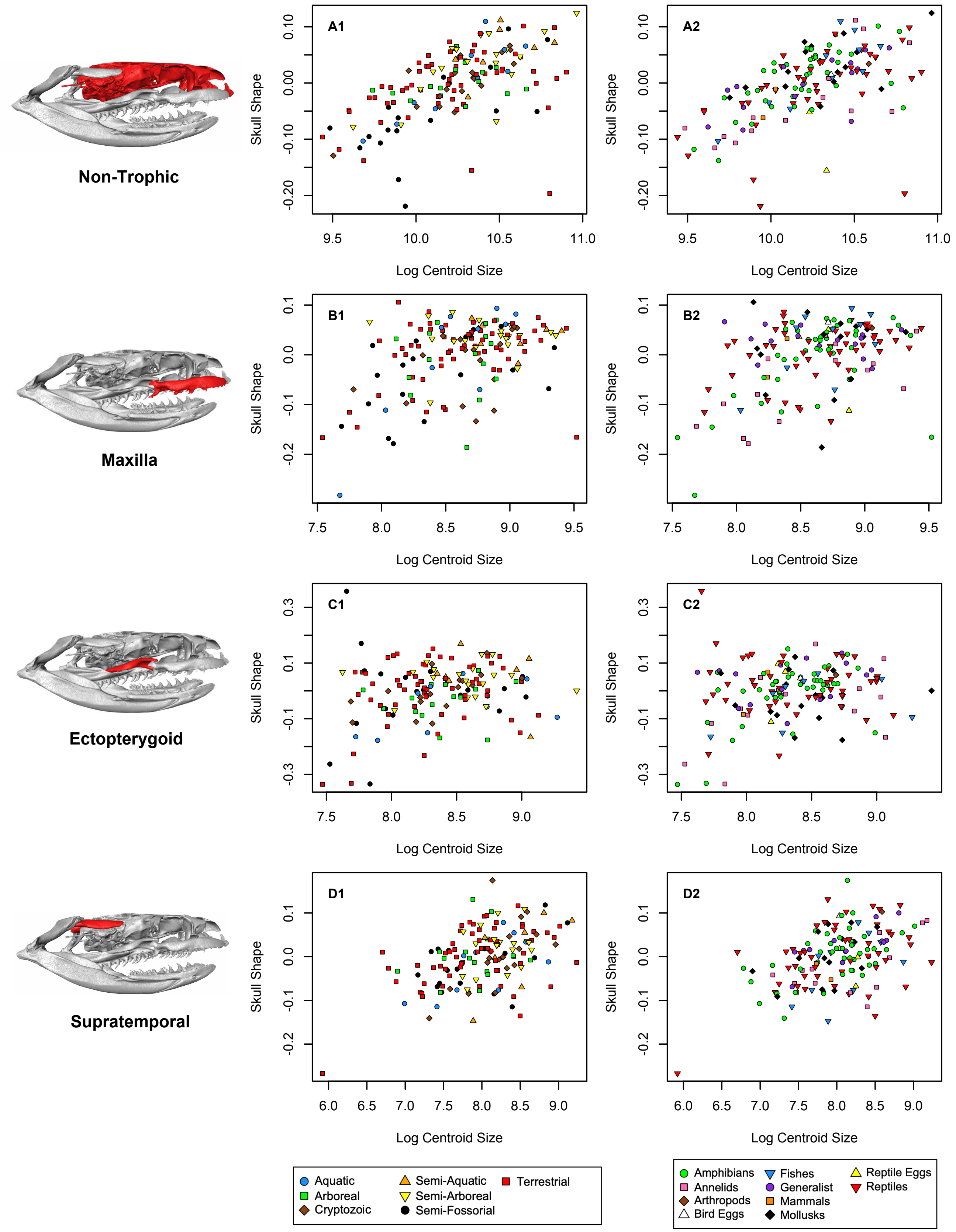


**Fig. S6. Allometry plots (regression of size against shape) in four modules: non-trophic (A), maxilla (B), ectopterygoid (C), and supratemporal (D) with respect to habitat use (1) and primary diet (2).** Allometry appears to be the strongest in the non-trophic module, with looser allometric relationships visible in the maxilla and supratemporal. A rough pattern of isometry is visible in the ectopterygoid.

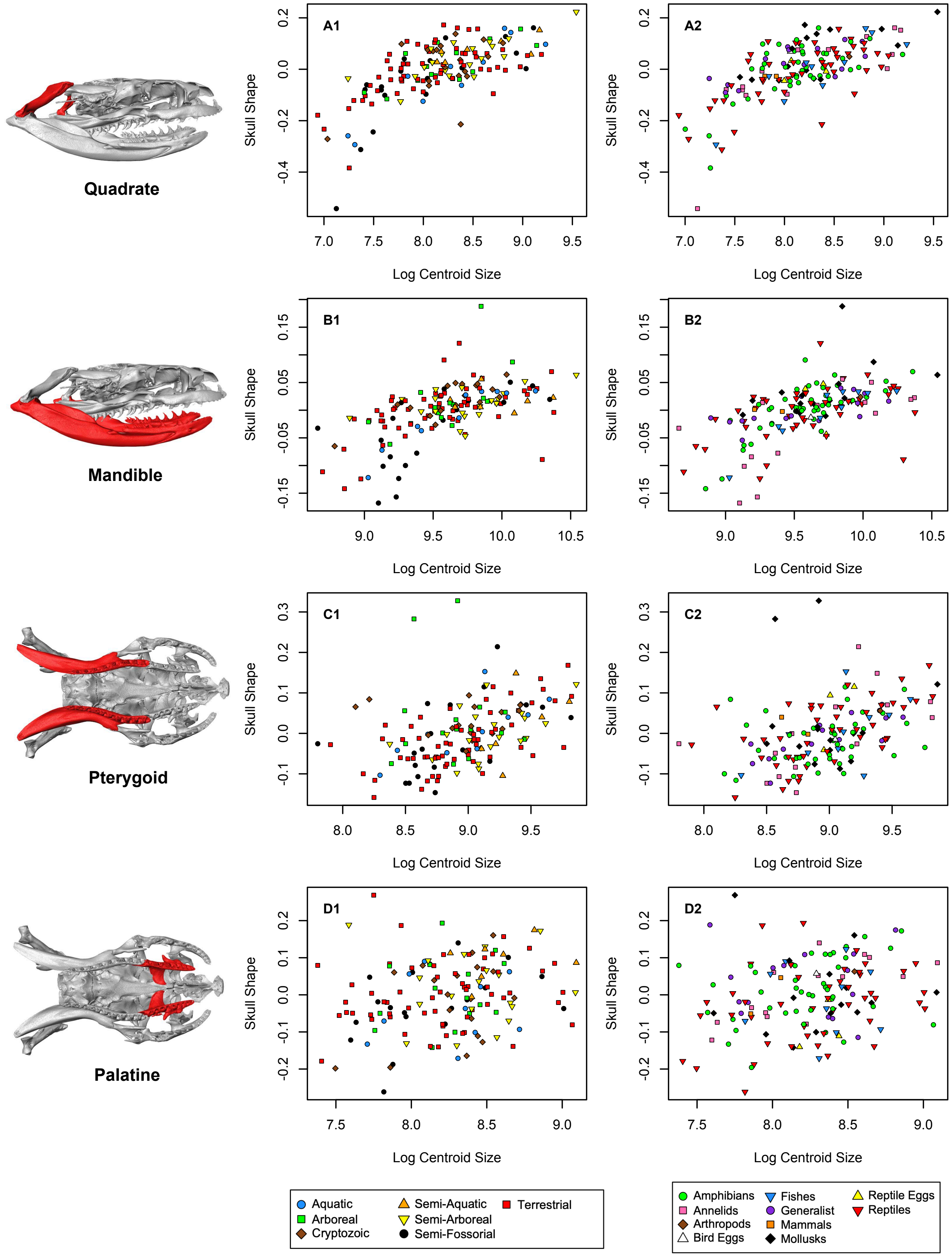


**Fig. S7. Allometry plots (regression of size against shape) in four modules: quadrate (A), mandible (B), pterygoid (C), and palatine (D) with respect to habitat use (1) and primary diet (2).** Allometry appears to be the strongest in the quadrate and mandible, with a looser pattern between size and shape in the pterygoid and palatine.
